# Acid Dissociation Constant and Related Thermodynamic Quantities for Triethanolammonium Ion in Water From 0 to 50°C^1^

**DOI:** 10.6028/jres.064A.033

**Published:** 1960-08-01

**Authors:** Roger G. Bates, Guy F. Allen

## Abstract

Earlier studies of the dissociation constants of monoethanolammonium and diethanolammonium ions and the thermodynamic constants for the dissociation processes have been supplemented by a similar study of triethanolammonium ion from 0° to 50° C. The dissociation constant (*K_bh_*) is given by the formula

$$-\log\;K_{bh}=1341.16/T+4.6252-0.0045666T$$

where *T* is in degrees Kelvin. The order of acidic strengths of the ions is as follows: Triethanolammonium >diethanolammonium>monethanolammonium. Conversely, monoethanolamine is the strongest of the three bases. The thermodynamic constants for the dissociation of one mole of triethanolammonium ion in the standard state at 25° C are as follows: Heat content change (Δ*H*°) 33.450 joule mole^−1^; entropy change (Δ*S*°), −36.4 joule deg^−1^ mole^−1^; heat-capacity change $\left(\Delta C_{p}^{{}^{\circ}}\right)$, 52 joule deg^−1^ mole^−1^.

## 1. Introduction

The dissociation of positively charged weak acids is an isoelectric process, occurring without the creation of new electrostatic charges. There should therefore be no significant electrostatic contribution to the change of heat capacity that accompanies the dissociation. Hence, the thermodynamic constants for dissociation processes of this charge type may be expected to reveal information concerning the nature of the specific chemical interactions that occur between the solvent and the dissolved molecules and ions. In addition, the strengths of organic bases provide a useful insight into the inductive (electron-donating and electron-attracting) powers of substituent groups.

In earlier work [1, 2],^2^ the substitution of hydroxyl groups into the substituents of alkylammonium ions was found to reduce the magnitude of the heat-capacity change occurring when 1 mole of the ion dissociates in the standard state to form hydrogen ion and 1 mole of the corresponding ammonia base. Following Everett and Wynne-Jones [3], this result was attributed to a reduction by hydroxyl of the hydrophobic character of the alkyl group. As a consequence of the electron-attracting property of the hydroxyl group, however, the monoethanolamine and diethanolamine are considerably weaker bases than ethylamine and diethylamine.

The study of the dissociation of substituted ammonium ions has now been extended to triethanolammonium. The acidic dissociation constant of this positively charged acid has been determined by electromotive-force measurements at intervals of 5°C from 0° to 50° C. Earlier studies [4, 5] of the dissociation constant of triethanolammonium were confined to a narrow range of temperatures. The standard changes of heat content, entropy, and heat capacity accompanying the dissociation process have been computed from the temperature coefficient of the dissociation constant. As expected, triethanolamine is a weaker base than diethanolaniine, which is, in turn, weaker than monoethanolamine.

## 2. Method

The electromotive force method and many of the experimental techniques have been described in detail elsewhere [1, 6, 7]. The cell used is represented schematically as follows:

$$\begin{array}{l} \text{Pt};H_{2}\left(g\right),{\left({\text{HOC}}_{2}H_{4}\right)}_{3}N\cdot \text{HCl}\;\left(m_{1}\right),\\ \;{\left({\text{HOC}}_{2}H_{4}\right)}_{3}N\;\left(m_{2}\right),\;\text{AgCl};\text{Ag}. \end{array}$$

The dissociation process can be formulated most simply,

$${\text{BH}}^{+}=B+H^{+},$$ (1)

where BH^+^ and B represent, respectively, triethanolammonium ion and triethanolamine. The complete expression by which the acidic dissociation constant, *K_bh_*, was determined is

$$-\log\;K_{bh}^{\prime }\equiv -\log\;K_{bh}-\beta m_{1}=pwH+\log\;\frac{m_{1}}{m_{2}}-\frac{2A\surd m_{1}}{1+Ba*\surd m_{1}}.$$ (2)

The hydrogen ion function *pw*H is derived from the emf (*E*) of the cell without liquid junction by the formula

$$pwH\equiv -\log\;\left(f_{H}f_{C1}m_{H}\right)=\left(E-E{}^{\circ}\right)F/\left(2.3026RT\right)+\log\;m_{1},$$ (3)

where *E*° is the standard potential of the cell [8], *m* is molality, *f* is an activity coefficient on the molal scale, and the other symbols have their usual significance.

The following steps in the derivation of eq (2) should be noted:

1. The mass-law expression for eq (1) is combined with eq (3).
2. The equilibrium concentrations of BH^+^ and B are set equal to *m*_1_ and *m*_2_, respectively, in view of the fact that neither species is a strong enough acid or base to be appreciably solvolyzed.
3. The variation of the activity coefficient term *f*_BH_^+^*f*_C1−_/*f*_B_ as a function of ionic strength is expressed by an equation of the Hückel form containing two parameters, a* and *β*, and the Debye-Hückel constants, *A* and *B* [9].
4. The ionic strength of each solution is equal to the molality of triethanolammonium chloride (*m*_1_).

## 3. Procedures and Results

Hydrochloric acid of reagent grade was diluted to about 6*N* and distilled in an all-glass still; the middle third was collected and redistilled. The twice-distilled acid was diluted to form solutions of molality about 0.1. These stock solutions were standardized gravimetrically by the silver chloride method. Colorless triethanolamine of the best commercial grade was distilled three times in vacuum, the middle fraction of the distillate being retained. The purified product was assayed by titration with the standard solution of hydrochloric acid. The assay value was 100.07 percent of the theoretical figure.

The cell solutions were prepared either by: (1) Adding weighed quantities of the pure triethanolamine to a solution of hydrochloric acid, the molar quantity of amine being approximately twice the number of moles of hydrochloric acid present; or (2) diluting with distilled water solutions prepared in this manner. Before the cells were filled, dissolved air was removed from the buffer solutions by bubbling purified hydrogen through them. Precautions were taken to prevent changes of concentration from occurring as a result of the deaeration.

The solubility of silver chloride in the cell solutions was so low that separation of the electrode compartments and corrections for solubility [6] were unnecessary, as attested by the absence of a gray deposit of silver on the platinum electrode at the conclusion of a run. The temperature of the water bath was measured by a calibrated mercury-in-glass thermometer. It was known to ±0.02° C.

**Figure 1:**
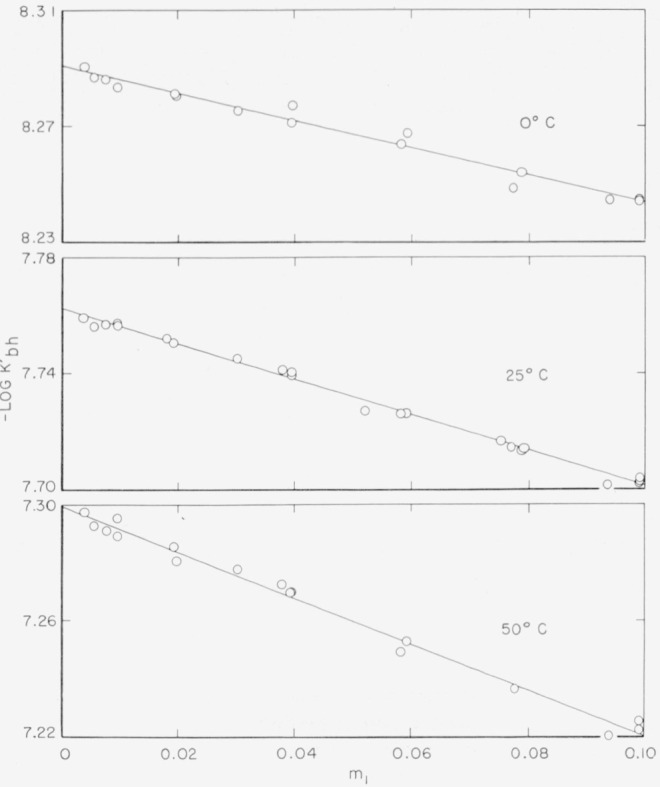
Plots of $-\log\;K_{\text{bh}}^{\prime }$ as a function of m_1_ at 0°, 25°, and 50° C.

The emf data were corrected in the usual manner to 1 atm partial pressure of gaseous hydrogen, and pwH was calculated by eq (3). Each value of pwH given in table 1 is the average of the results obtained from two hydrogen-silver chloride electrode combinations in the same cell. Values of $-\log\;K_{bh}^{\prime }$ were calculated by eq (2) for several values of the parameter a* and were plotted as a function of *m*_1_. Straight-line plots were obtained at each temperature when a*=0 was chosen, as shown in figure 1. The true −log *K_bh_*, the intercept of these lines at *m*_1_ = 0, was obtained by the method of least squares.

The values of −log *K_bh_* are summarized in table 2, together with the standard deviations (S.D.) of the intercepts. The last two columns of table 2 list −log *K_b_* and *K_b_*, where *K_b_* is the basic dissociation constant of triethanolamine obtained from *K_bh_* and *K_w_* the ion-product constant of water [7]. by the formula

$$K_{b}=K_{w}/K_{bh}.$$ (4)

The value of −log *K_bh_* at 25° C (7.762) is to be compared with 7.77 found by Hall and Sprinkle [4] and by Bates and Schwarzenbach [5].

## 4. Thermodynamic Quantities

The values of −log *K_bh_* given in Table 2 were fitted to an equation of the Harned-Robinson form [10] by the method of least squares. Between 0° and 50° C, *K_bh_* is given by the expression

$$-\log\;K_{bh}=1341.16/T+4.6252-0.0045666T$$ (5)

where *T* is the temperature in deg Kelvin. The average difference between the “observed” *K_bh_* at the 11 temperatures and that calculated by eq (5) is 0.0009 unit.

The changes of Gibbs free energy (Δ*G*°), of enthalpy (Δ*H*°), of entropy (Δ*S*°), and of heat capacity $\left(\Delta C_{p}^{{}^{\circ}}\right)$ for the dissociation of 1 mole of triethanolammonium ion in the standard state were computed from the constants of eq (5) by the following formulas:

$$\Delta G{}^{\circ}=2.3026R\left(A+BT+CT^{2}\right),$$ (6)

$$\Delta H{}^{\circ}=2.3026R\left(A-CT^{2}\right),$$ (7)

ΔS°=2.3026R(−B−2CT), (8)

$$\Delta C_{p}^{{}^{\circ}}=2.3026R\left(-2CT\right).$$ (9)

The values of *A*, *B*, and *C* are, respectively, 1341.16, 4.6252, and −0.0045666. The results are summarized in Table 3. From the standard deviation of log *K_bh_*, the uncertainties in the thermodynamic quantities at 25° C are estimated to be as follows: Δ*G*°, 6 j mole^−1^; Δ*H*°, 100 j mole^−1^; Δ*S*°, 0.5 j deg^−1^, mole^−1^; and $\Delta C_{p}^{{}^{\circ}}$, 5 j deg^−1^ mole^−1^.

## 5. Discussion

The strengths of acids and bases are influenced by polar (inductive) and resonance effects within the molecule, by the possibility of internal hydrogen bonding, and steric factors. It is, however, difficult to impede the addition or removal of a proton by the addition of bulky groups to the molecule. Furthermore, resonance and intramolecular hydrogen bonding do not usually play an important role in the dissociation of the simple aliphatic substituted ammonias. In the Lewis concept, the strength of nitrogen bases is a measure of the availability of a donor electron pair, and the effect of polar substituent groups on the basic strength can sometimes be satisfactorily accounted for in a qualitative way by inductive influences. The electron-attracting properties of the hydroxyl group are no doubt largely responsible for the fact that the ethanolamines are weaker bases than the corresponding ethylamines.

The dissociation of a weak base or acid is, however, fundamentally a protolytic process involving both the acid (or base) in question and the solvent as well. It is quite understandable, therefore, that the extent to which such a reaction proceeds should depend not only on the intrinsic acidic or basic strengths of the two reacting species but also upon other factors which either limit or enhance the probability that the reacting species will approach so closely that reaction is favored. In this connection, it is well to bear in mind that solvation may well be the initial stage of acidic or basic dissociation. Entirely apart from electronic effects, therefore, dissociation in water may be favored or hindered by the size and shape of the acid or base molecule (steric factors) and by its hydrophilic or hydrophobic character (chemical factors).

When a monobasic cationic acid such as triethanolammonium (BH^+^) dissociates, the process is perhaps best regarded as the separation of solvated BH^+^ ions into free amine and hydronium ions (H_3_O^+^). Inasmuch as the amine is uncharged it is presumably not highly effective in orienting the polar water molecules. Hence, the degree of solvation may be strongly influenced by steric and chemical factors which are relatively unimportant with charged species. In the dissociation process water molecules may therefore be released from combination.

These effects are likely to be reflected in the values of the entropy and heat-capacity changes for the dissociation process; release of water molecules should result in an increase of entropy and heat capacity. Any factor, steric or hydrophobic for example, tending to exclude solvent molecules and reduce solvation of the free amine would therefore be expected to make the entropy and heat capacity changes for the process more positive (less negative).

Considerations of this sort led Everett and Wynne-Jones [3] to ascribe the positive heat-capacity change in the dissociation of the methyl-substituted ammonium ions (as compared with $\Delta C_{p}^{{}^{\circ}}=0$ for ammonium ion [3, 6] to the hydrophobic character of alkyl groups. For the dissociation of monoethanolammonium ion, $\Delta C_{p}^{{}^{\circ}}$ is about −5 j deg^−1^ mole^−1^ [1] and for diethanolammonium ion about +49 j deg^−1^ mole^−1^ [2], as compared with +52 j deg^−1^ mole^−1^ given in table 3 for triethanolammonium ion. These results suggest that increasing substitution of ethanol groups into ammonia has two effects, namely (1) progressively decreasing hydrophobic character (tending to lower $\Delta C_{p}^{{}^{\circ}}$), and (2) progressively greater steric hindrance (tending to raise $\Delta C_{p}^{{}^{\circ}}$ by exclusion of solvent). Likewise, a contrary variation of the entropy change and the heat-capacity change, although observed heretofore, has not, to the authors knowledge, yet been satisfactorily explained. A more illuminating comparison than this one could doubtless be made between the ethanolamines and the corresponding ethyl amines, but unfortunately the heat-capacity data needed are unavailable.

## Figures and Tables

**Table 1:** pwH at 0° to 50° C for buffer solutions composed of triethanolammonium hydrochloride (*m*_1_) and triethanolamine (*m*_2_)

*m*_1_	*m*_2_	0°	5°	10°	15°	20°	25°	30°	35°	40°	45°	50°
												
0.09909	0.10481	8.5764	8.4611	8.3569	8.2479	8.1453	8.0473	7.9494	7.8534	7.7615	7.6727	7.5848
.09908	.14403	……….	8.5994	8.4912	8.3857	8.2832	8.1851	……….	……….	……….	……….	……….
.09908	.10765	8.5882	8.4722	8.3643	8.2589	8.1566	8.0604	7.9599	7.8665	7.7694	7.6784	7.5932
.09379	.09312	8.5408	8.4281	8.3184	8.2112	8.1079	8.0102	7.9113	7.8191	7.7257	7.6375	7.5431
.07923	.08381	……….	8.4363	8.3297	8.2257	8.1239	8.0250	7.9281	7.8325	7.7402	7.6518	……….
.07896	.08578	8.5643	8.4460	8.3356	8.2319	8.1315	8.0353	7.9321	7.8357	……….	……….	……….
.07707	.07651	8.5166	8.4056	8.2979	8.1920	8.0911	7.9940	7.8950	7.7987	7.7059	7.6153	7.5286
.07522	.10934	……….	8.5737	8.4641	8.3587	8.2563	8.1582	……….	……….	……….	……….	……….
.05928	.06440	8.5415	8.4219	8.3151	8.2109	8.1087	8.0097	7.9084	7.8184	7.7235	7.0340	7.5479
.05830	.06166	8.5240	8.4067	8.3001	8.1951	8.0938	7.9962	7.8967	7.8019	7.7089	7.6192	7.5304
.05215	.05178	……….	8.3681	8.2606	8.1562	8.0559	7.9563	7.8612	7.7664	……….	……….	……….
.03952	.04294	8.5072	8.3905	8.2831	8.1799	8.0782	7.9787	7.8803	7.7889	7.6930	7.6041	7.5170
.03949	.04177	8.4897	8.3771	8.2706	8.1662	8.0651	7.9658	7.8686	7.7740	7.6813	7.5926	7.5054
.03797	.05520	……….	8.5107	8.4044	8.3001	8.1979	8.1020	8.0022	7.9072	7.8162	7.7263	7.6422
.03039	.03018	8.4426	8.3290	8.2227	8.1188	8.0179	7.9192	7.8208	7.7270	7.6354	7.5459	7.4600
.019780	.02029	8.4423	8.3279	8.2214	8.1176	8.0161	7.9198	7.8210	7.7239	7.6358	7.5432	7.4546
.019283	.02095	8.4529	8.3367	8.2304	8.1269	8.0254	7.9279	7.8310	7.7396	7.6445	7.5557	7.4692
.009568	.010120	8.4033	8.2878	8.1857	8.0789	7.9779	7.8806	7.7846	7.6906	7.5992	7.5094	7.4237
.009541	.013869	……….	8.4273	8.3212	8.2175	8.1146	8.0191	……….	……….	……….	……….	7.5560
.007774	.008223	8.3967	8.2796	8.1734	8.0697	7.9668	7.8710	7.7750	7.6793	7.5835	7.4974	7.4091
.005557	.005877	8.3841	8.2657	8.1599	8.0563	7.9556	7.8566	7.7623	7.6673	7.5752	7.4855	7.3964
.003869	.004092	8.3757	8.2584	8.1533	8.0497	7.9483	7.8469	7.7547	7.6586	7.5652	7.4740	7.3880

**Table 2:** Summary of values for *K_bh_* and *K_b_*

*t*	−log *K_bh_*	*S.D.*	*K_bh_×*10^8^	−log *K_b_*	*K_b_×*10^7^
					
*° C*					
0	8.290_6_	±0.0009	0.512	6.652	2.23
5	8.173_4_	.0007	.671	6.561	2.75
10	8.067_4_	.0009	.936	0.468	3.40
15	7.963_2_	.0008	1.088	6.383	4.14
20	7.861_1_	.0007	1.377	6.306	4.94
25	7.762_4_	.0006	1.728	6.234	5.83
30	7.666_1_	.0009	2.16	6.167	6.81
35	7.570_7_	.0010	2.69	6.109	7.78
40	7.477_3_	.0008	3.33	6.058	9.45
45	7.387_5_	.0009	4.10	6.008	9.82
50	7.299_2_	.0012	5.02	5.963	10.89

**Table 3:** Thermodynamic quantities for the dissociation of 1 mole of triethanolammonium ion in the standard state

*t*	Δ*G*°	Δ*H*°	Δ*S*°	$\Delta C_{p}^{{}^{\circ}}$
				
° *C*	*j mole*^−1^	*j mole*^−1^	*jdeg*^−1^ *mole*^−1^	*jdeg*^−1^ *mole*^−1^
0	43,340	32,200	−40.8	48
5	43,422	32,440	−39.9	49
10	43,740	32,690	−39.0	50
15	43,932	32,940	−38.2	50
20	44,121	33,190	−37.3	51
25	44,305	33,450	−36.4	52
30	44,485	33.710	−35.5	53
35	44,608	33,980	−34.7	54
40	44,832	34,250	−33.8	55
45	44,999	34,530	−32.9	56
50	45,161	34,810	−32.0	56

## Footnotes

2 Figures in brackets indicate the literature references at the end of this paper.
